# Pharmacological Inhibition of Class III Alcohol Dehydrogenase 5: Turning Remote Ischemic Conditioning Effective in a Diabetic Stroke Model

**DOI:** 10.3390/antiox11102051

**Published:** 2022-10-18

**Authors:** Syed Kashif Zaidi, Md Nasrul Hoda, Shams Tabrez, Mohammad Imran Khan

**Affiliations:** 1 Center of Excellence in Genomic Medicine Research, Faculty of Applied Medical Sciences, King Abdulaziz University, Jeddah 21589, Saudi Arabia; 2 Department of Neurology, Henry Ford Health System, Detroit, MI 48202, USA; 3 King Fahd Medical Research Center, King Abdulaziz University, P.O. Box 80216, Jeddah 21589, Saudi Arabia; 4 Medical Laboratory Sciences, Faculty of Applied Medical Sciences, King Abdulaziz University, Jeddah 21589, Saudi Arabia; 5 Department of Biochemistry, Faculty of Life Science, King Abdulaziz University, Jeddah 21589, Saudi Arabia; 6 Center of Artificial Intelligence for Precision Medicines, King Abdulaziz University, Jeddah 21589, Saudi Arabia

**Keywords:** stroke, remote ischemic conditioning, nitric oxide synthase 3, alcohol dehydrogenase 5, ADH5, *s*-nitrosoglutathione, GSNO, GSNO reductase, GSNOR

## Abstract

The restoration of cerebral blood flow (CBF) to achieve brain tissue oxygenation (PbtO_2_) is the primary treatment for ischemic stroke, a significant cause of adult mortality and disability worldwide. Nitric oxide (NO) and its bioactive *s*-nitrosylated (SNO) reservoirs, such as *s*-nitrosoglutathione (GSNO), induce hypoxic vasodilation to enhance CBF during ischemia. The endogenous pool of SNOs/GSNO is enhanced via the activation of endothelial NO synthase (eNOS/NOS3) and by the suppression of class III alcohol dehydrogenase 5 (ADH5), also known as GSNO reductase (GSNOR). Remote ischemic conditioning (RIC), which augments NOS3 activity and SNO, is an emerging therapy in acute stroke. However, RIC has so far shown neutral effects in stroke clinical trials. As the majority of stroke patients are presented with endothelial dysfunctions and comorbidities, we tested the hypothesis that NOS3 dysfunction and diabetes will abolish the protective effects of RIC therapy in stroke, and the prior inhibition of GSNOR will turn RIC protective. Our data demonstrate that RIC during thrombotic stroke failed to enhance the CBF and the benefits of thrombolysis in NOS3 mutant (NOS3^+/−^) mice, a genetic model of NOS3 dysfunction. Interestingly, thrombotic stroke in diabetic mice enhanced the activity of GSNOR as early as 3 h post-stroke without decreasing the plasma nitrite (NO_2_^−^). In thrombotic stroke, neither a pharmacological inhibitor of GSNOR (GRI) nor RIC therapy alone was protective in diabetic mice. However, prior treatment with GRI followed by RIC enhanced the CBF and improved recovery. In a reperfused stroke model, the GRI–RIC combination therapy in diabetic mice augmented PbtO_2_, a translatory signature of successful microvascular reflow. In addition, RIC therapy unexpectedly increased the inflammatory markers at 6 h post-stroke in diabetic stroke that were downregulated in combination with GRI while improving the outcomes. Thus, we conclude that preexisting NOS3 dysfunctions due to comorbidities may neutralize the benefits of RIC in stroke, which can be turned protective in combination with GRI. Our findings may support the future clinical trial of RIC in comorbid stroke. Further studies are warranted to test and develop SNO reservoirs as the blood-associated biomarker to monitor the response and efficacy of RIC therapy in stroke.

## 1. Introduction

Acute ischemic stroke, a major public health issue worldwide, often occurs (~87%) due to thromboembolic occlusion of the cerebral arteries [1]. Therefore, in order to reinstate cerebral blood flow (CBF) and brain tissue oxygenation (PbtO_2_), thrombolysis, with the gold-standard therapy in stroke, recombinant tissue plasminogen activator (rt-PA), and the recently approved mechanical thrombectomy, remain the key approaches to treating ischemic strokes [1,2]. On the other hand, freely diffusible nitric oxide (NO) during hypoxia or ischemia modulates the collateral microcirculatory flow via hypoxic vasodilation to enhance PbtO_2_ and neurovascular protection [3,4,5]. However, a recent clinical trial of exogenous nitrite (NO_2_^−^)/nitrate (NO_3_^−^) therapy in stroke demonstrated no benefits [6], while the risk of blood pressure (BP) lowering remains warranted. Therefore, the enhancement of alternate “endogenous” NO reservoirs remain appealing in stroke [6].

Endothelial NO synthase (eNOS or NOS3) activity in the endothelium and red blood cells (RBCs) primarily constitutes the pool of vasculo-humoral NO reservoirs which protects against ischemic injury [7,8]. The genetic deletion of NOS3 augmented microvascular dysfunctions and brain tissue hypoxia in aged mice and also exacerbated acute stroke injury in young mice, establishing the critical role of endothelial NO in neurovascular protection [2,7,8,9,10]. On the other hand, class III alcohol dehydrogenase 5 (ADH5), also known as *s*-nitrosoglutathione reductase (GSNOR), catabolizes and depletes the pool of major *s*-nitrosylated (SNO) reservoirs of endogenous NO, such as the small molecule NO carrier, *s*-nitrosoglutathione (GSNO) [11]. We reported that exogenous GSNO therapy in thrombotic stroke improved the CBF, PbtO_2_, and behavioral outcomes while reducing the stroke injury volume in diabetic mice [12]. Alternatively, the genetic deletion and pharmacological inhibition of GSNOR also protected against ischemia-reperfusion (IR) injury as a result of increased endogenous SNO/GSNO [13,14]. GSNOR inhibitor (GRI) preserved the vasculoprotective benefits of a low level of endogenous NO in hypertension, one of the common comorbidities associated with aging and stroke [15]. GRI therapy in hypertensive rats with impaired NO production preserved the flow-mediated dilation (FMD) and protected against microvascular and conduit artery dysfunctions [15]. GRI therapy also protected against the mechanical cerebral IR injury in mice with NOS3 dysfunction [14]. However, the effect of stroke on GSNOR in thromboembolic stroke and the benefits of its modulation under the varying dynamics of reperfusion and in combination with therapies in trials remain understudied.

Different modes of ischemic conditioning (IC), including remote ischemic conditioning (RIC) therapy, are being tested as enhancers of endogenous NO in acute IR injuries [16,17,18]. RIC therapy, a non-invasive procedure remote to the actual site of injury, is performed with the inflation–deflation of a pair of BP cuffs on the extremities (limbs). The resulting intermittent sub-lethal IR protects against the lethal IR via NOS3 activation and the subsequently increased endogenous NO metabolomes [12,18,19,20]. We and others have reported that RIC following stroke enhances CBF and improves the benefits of late intravenous thrombolysis (IVT) in rodents without comorbidities [20,21,22]. Sun et al. reported that IC preferentially increases SNO to protect against ischemia [23]. In contrast, it has also been reported that hyperglycemia abolishes the benefits of IC [24,25,26]. However, to the best of our knowledge, there are no reports of testing RIC therapy in diabetic stroke.

Diabetic subjects often suffer impaired NOS3 activity and remain at the higher risk of stroke and poor outcomes [27,28,29]. Thus, the resulting vascular dysfunctions and the complexity of diabetes enhance the risk of vascular fragility, which may depreciate the benefits of reperfusion, though controlling the hyperglycemia remains beneficial. Notably, glycosylated hemoglobin (Hb) in diabetes becomes a preferred target for vascular NO, producing tightly bounded NO [30], thereby likely reducing the overall delivery of bioactive NO in diabetic vasculature. As such, the *s*-nitrosylation of healthy (non-glycosylated) Hb enhances the carriage, delivery, bioactivity, and efficacy of NO in mitigating ischemic injuries via hypoxic vasodilation and adaptive collateralization of the vasculature [3,4,5,13], a physiological mechanism also implicated in the benefits from RIC [22].

In this preclinical trial of RIC and GRI therapies in murine models of stroke, we tested the hypothesis that reduced NOS3 would abolish the benefits of both therapies. We also tested the hypothesis that prior treatment with GRI in diabetic stroke would turn RIC therapy protective.

## 2. Materials and Methods

### 2.1. Animals, Experimental Groups, and Approaches

Male mice with either B6/129.NOS3 heterozygous mutants (NOS3^+/−^) or C57BL/6 wild-type (WT) backgrounds were obtained from our in-house colonies and were used in the stroke studies, following the approved protocols of the ethical committee of the Center of Excellence in Genomic Medicine Research (CEGMR) at King Abdulaziz University in accordance with the guidelines of the National Bioethical Committee of Saudi Arabia (NACSA) (approval code: 014-CEGMR-Eth-2). The effect of RIC therapy on the CBF before and after IVT in a thrombotic stroke model was first tested in NOS3^+/−^ mice, a genetic model of preexisting NOS3 impairment. Next, we determined the effect of thrombotic stroke on the plasma NO_2_^−^, plasma ADH5/GSNOR activity, and the activity and expression of ADH5 in brain microvascular endothelial cells (BMECs) in diabetic mice. Finally, we tested the efficacy of the RIC, GRI, and a combination of both therapies in diabetic mice subjected to either thrombotic or transiently occluded–reperfused stroke models.

### 2.2. Streptozotocin (STZ)-Induced Model of Type 1 Diabetes

Diabetes with STZ injection was performed as reported earlier [12]. Briefly, C57BL/6 male WT mice (10 ± 1 weeks old) were fasted overnight and injected with the first intraperitoneal (IP) dose of 100 mg/kg of STZ injection (Sigma Millipore Chemical Co., St. Louis, MO, USA), followed by once-daily IP injection of 50 mg/kg of STZ for 5 consecutive days. About 4 weeks apart from the beginning of the STZ injection, the diabetic mice (blood glucose levels ranging between 280 and 400 mg/dL) were used in the studies.

### 2.3. Models of Thrombotic and Reperfused Strokes

#### 2.3.1. Photothrombotic (PT) Model of Stroke

The fibrin-rich modified model of PT stroke was conducted as reported earlier [12,31] in mice injected with the analgesic buprenorphine SR (2 mg/kg) and anesthetized with isoflurane (1.5–2.5%). Briefly, the right skull bone above the zygomatic arc was exposed, and the mice were IV injected via the tail vein with an admixture of thrombin and rose bengal (RB) dye (80 U/kg and 50 mg/kg, respectively, in 200 μL of sterile saline). The distal trunk of the middle cerebral artery (MCA), visualized with the laser light, was illuminated for 15 min with a 532 nm laser light (4.5 mW; beam diameter 2.5–3 mm; Thorlabs, Newton, NJ, USA) to induce a thrombotic occlusion. The mice were randomized to the experimental groups after stroke.

#### 2.3.2. Reperfused Transient MCA Occlusion (tMCAO) Model of Stroke

A tMCAO stroke model in rodents resembles the IR events as it evolved in the clinical cases of stroke subjected to endovascular thrombectomy (EVT) for mechanical clot retrieval and subsequent large vessel reperfusion. The surgical procedure for the tMCAO stroke model was similar to the thromboembolic stroke model, as reported earlier [20,32]. However, instead of a clot, the transient IR was performed with a suture occlusion for 60 min. followed by withdrawal of the suture to achieve the large vessel reperfusion. Briefly, the right common carotid artery (CCA), the external carotid artery (ECA), and the internal carotid artery (ICA) were exposed with blunt dissections in mice under isoflurane. A 6-0 monofilament nylon suture with a ~3 mm silicone-coated tip (Doccol Corp., Sharon, MA, USA) was introduced into the ICA via an ECA stump. The suture was gently advanced into the MCA region until it was wedged to occlude the proximal stem of the MCA. The arteriotomy on the ECA stump was ligated after suture withdrawal to prevent bleeding.

### 2.4. Treatments and Therapies after Stroke

Wherever needed, non-invasive RIC therapy or RIC mock (sham procedure for RIC) was performed using a programmable RIC instrument (Hatteras Instruments, Cary, NC, USA) at 2 h after stroke, as reported earlier but with slight modification [20]. Briefly, BP cuffs for rodents were put around the two hind limbs of the mice under isoflurane anesthesia. The programmable instrument was set to 5 cycles of BP cuff inflation × 5 min of inflation at 200 mm Hg × 5 min interval between cycles following deflation. GRI therapy (N-6022; Axon Medchem, Reston, VA, USA) was IV injected via the tail vein (2.5 mg/kg) at 1 h after thrombotic occlusion in the PT stroke model and at 10 min before reperfusion in the tMCAO model of reperfused stroke. Delayed IVT therapy was given as rt-PA (5 mg/kg; CathFlow, Genentech, San Francisco, CA, USA) infusion at 4 h post-stroke, as reported earlier [12,20]. All sham operation (sham-op) groups for the therapies were either infused with the vehicle (for GRI and IVT therapies) or underwent the RIC mock for the sham-operated procedure of RIC therapy. The sham groups for combination therapy controls received both the vehicle and the RIC mock.

### 2.5. Measurement of Relative Cerebral Blood Flow (CBF)

Cortical laser doppler flowmetry (LDF; Perimed. Inc., Jarfalla, Sweden) was used to confirm the occlusion of cerebral vessels and focal drop in the CBF in anesthetized mice, as reported earlier [12,33]. Laser speckle contrast imaging (LSCI) analysis (LASCA) was also performed as reported earlier [12,20,34]. Briefly, the anesthetized mice with exposed skulls were scanned using an RFLSI-III LSCI instrument (RWD Life Sciences, San Diego, CA, USA). The perfusion values from the acquired images of the ipsilateral ischemic hemisphere were normalized with the values from the contralateral uninjured hemisphere. Relative CBF was calculated and presented as the % change to that of contralateral side.

### 2.6. Quantification of Hemoglobin (Hb) Content

Male NOS3^+/−^ mice, treated with or without RIC and delayed IVT therapies, were euthanized under deep anesthesia (isoflurane > 4.5%) at 6 h post-stroke after the measurement of CBF and were perfused with 50 mL of chilled 1× PBS to harvest their brains. The Hb content in the ipsilateral hemispheres was quantified using Drabkin’s reagent (Sigma Millipore Chemical Co., St. Louis, MO, USA), as reported earlier [12,20].

### 2.7. Analysis of Plasma Nitrite (NO_2_^−^) Level

Citrated blood samples from the diabetic mice, with or without (sham) stroke, were collected via cardiac puncture at 3 h post-stroke to separate the plasma. The plasma NO_2_^−^ level (*n* = 10 mice/group) was measured using a commercial kit and by following the manufacturer’s protocol (Cat# 780001; Cayman Chemical Co., Ann Arbor, MI, USA). These diabetic mice also were perfusion sacrificed with 50 mL of chilled 1× PBS to harvest the ipsilateral hemispheres for the enrichment of the BMECs, as described below. GSNOR activity in the remaining plasma samples (*n* = 10 mice/group) and the BMECs (*n* = 6 mice/group) and the GSNOR expression in the BMECs (*n* = 4 mice/group) were performed as described below.

### 2.8. Isolation and Enrichment of Mouse BMECs

The mouse BMECs were enriched as reported earlier [35,36]. Briefly, the meninge-free ipsilateral hemispheres were minced and digested with collagenase–DNAse–dispase mixtures to obtain a myelin-free pellet. The pellets, resuspended in 2 mL of Dulbecco’s Modified Eagle’s Medium (DMEM), were loaded on top of the 30% Percoll gradient (Sigma Millipore Chemical Co., St. Louis, MO, USA) and centrifuged at 4 °C and 700× *g* for 10 min to obtain an interphase content, which was centrifuged again at 4 °C/1000× *g* for 10 min to enrich the BMECs.

### 2.9. Estimation of GSNOR Activity in Plasma and BMECs

All the analytical grade chemical reagents used were obtained from Sigma Millipore Co., Saint Louis, MO, USA. A method of GSNO-dependent NADH consumption was used to determine GSNOR activity, as reported elsewhere [37,38]. Briefly, the BMEC samples were lysed in a buffer containing 50 mM Tris-HCl (pH 8.0), 150 mM NaCl, 1 mM EDTA, 0.1% Triton X-100, and 1:100 (*v*/*v*) protease inhibitor cocktail and centrifuged at 4 °C and 10,000× *g* for 10 min. Next, the plasma and brain tissue supernatants were diluted to a protein concentration of 1 mg/mL and 0.1 mg/mL, respectively, in a reaction buffer containing 20 mM Tris-HCl (pH 8.0), 0.5 mM EDTA, and 0.75 µM NADH. The samples were incubated in duplicate with or without 100 µM GSNO in a 96-well plate. The NADH consumption was monitored using a microplate reader (Ex/Em: 340/460 nm; Varioskn LUX, Thermo Fisher Scientific, Waltham, MA, USA). The GSNOR activity was calculated by subtracting the rate of NADH consumption with GSNO minus the rate without GSNO.

### 2.10. Immunoblot of GSNOR Expression in BMECs

For the GSNOR expression in the BMECs, routine gel electrophoresis followed by immunoblot was performed. Briefly, the BMECs in modified RIPA (radio-immunoprecipitation assay) lysis buffer (Upstate, Lake Placid, NY, USA), supplemented with 40 mM NaF, 2 mM Na_3_VO_4_, 0.5 mM phenylmethylsulfonyl fluoride (PMSF), and 1:100 (*v*/*v*) of proteinase inhibitor cocktail (Sigma Millipore Co., Saint Louis, MO, USA), were electrophoresed and immunoblotted against antibodies for anti-GSNOR (Rb mAb Cat# 177932, Abcam, Waltham, MA, USA) and alpha-tubulin (𝛼-Tub; Ms mAb Cat# HRP-66031, Proteintech, Rosemont, IL, USA) as the loading control. The immuno-densitometric signal for GSNOR was quantified in arbitrary units (AU) using the NIH’s free ImageJ software and was normalized with the corresponding 𝛼-tubulin signal.

### 2.11. Behavioral Tests for Motor Function and Neurologic Deficits

A beam walking test (BWT) was performed to assess motor function and balance as reported earlier [12]. Briefly, pre-trained mice were tested to walk for 3 trials × 5 min intervals between trials on a 100 cm × 5 mm narrow beam. The BWT performance was quantified in the context of the time taken (in seconds, s) to cross the beam and also as the number of foot slips/faults that occurred during the walk. The mean values of the above outcomes from 3 trials were calculated and presented as the BWT outcome measures [39,40]. The corner test, a well-recognized behavioral test to simultaneously assess sensorimotor function and postural asymmetry in animal models of unilateral brain injury, was performed as described elsewhere, with 10 trials and each trial at a 30 s time interval [41]. As such, rodents without unilateral injury do not show asymmetrical bias for turning around. However, animals with unilateral injury, such as those with unilateral cerebral ischemia injury, show increased ipsilateral preference and decreased contralateral turns. Briefly, the mice were individually introduced between the two boards (placed at an angle of 30°) such that they faced walking towards the corner. While entering the corner, vibrissae stimulations encouraged frequent turns to find the opportunities to exit the corner. The contralateral and ipsilateral turns from 10 trials were averaged, and the mean laterality index was calculated as the ratio of the contralateral vs. the ipsilateral turns. An increased ratio of laterality index is presented as the improved sensorimotor and postural symmetry outcome. Moreover, the rotarod test was performed using a Panlab System (Harvard Apparatus, Cambridge, MA, USA) with a 3 cm diameter, at a constant velocity of 15 rpm, a maximal time window of 60 s, and the mean value of the fall latency from 3 trials (each trial 5 min apart) as the functional outcome for motor coordination. The neurologic deficit score (NDS) on a 5-point modified Bederson NDS scale, after the tMCAO strokes in the mice, was assessed by an investigator blinded to the groups, as reported earlier [12,20]. The highest NDS number indicates the worst outcome while the lower number indicates reduced neurological deficit.

### 2.12. Measurement of Infarct Volume, Edema, and BBB Permeability

1,3,5-triphenyl tetrazolium chloride (TTC) staining was used to differentiate and determine the volume of metabolically inactive core (dead injured area) from that of the metabolically active live penumbra region [12,20], to calculate the infarct volume as the absolute infarct volume (mm^3^) in the PT stroke model of the mini-infarct and as the percent (%) corrected infarct volume normalized to the uninjured contralateral side in the tMCAO stroke model of the larger infarct volume with significant swelling. Moreover, edema was semi-quantitated as the % swelling in the tMCAO stroke model. A slightly modified method of IV injection of Evan’s blue (EB) dye, followed by 12 h of circulation time was used to assess the BBB integrity, as reported earlier [12,20].

### 2.13. Measurement of Brain Tissue Oxygenation (PbtO_2_)

PbtO_2_, using a polarographic micro-electrode, was measured as reported elsewhere [42,43,44], with a slight modification as adopted earlier for the fluorescence quenching method of the PbtO_2_ measurement [12]. The system was composed of a fast response modified Clark’s polarographic micro-electrode for oxygen (tip diameter 50 µm, 90% response time <2 s; Unisense, Denmark) integrated to an amplifying picoammeter (Unisense) and interfaced with dedicated software (SensorTrace, Unisense, Denmark). Following 2-point calibration for zero and ambient PO2 in 50 mL artificial cerebrospinal fluid (aCSF, please see Appendix A) containing either sodium sulfite or saturated with medical grade air, respectively, the oxygen sensor was inserted into the brain through a 1 mm burr hole in the skull on the stereotaxic coordinates (AP −1 mm, ML +1 mm, DV 2 mm from the dura). It was slightly retracted (~0.5 mm) to create a void space between the electrode tip and the brain tissue. After stabilizing the response, the PbtO_2_ was continuously sampled at 60 Hz for 3 min, and the recorded data were averaged to obtain the mean value of the PbtO_2_.

### 2.14. Semi-Quantitative Real-Time PCR (qPCR) for Inflammatory Gene Expressions

Total RNA from the brain tissue samples was extracted using a Direct-Zol RNA isolation kit (Zymo Research, Irvine, CA, USA), and the complementary deoxyribonucleic acid (cDNA) was prepared using the iScript™ cDNA synthesis kit (Bio-Rad, Hercules, CA, USA), following the manufacturer’s protocol and using a programmable thermal cycler (C1000™ Touch, Bio-Rad, Hercules, CA, USA). The qPCR assay was performed using an Iraq universal SYBER Green PCR master mix and the Bio-Rad iCycler iQ PCR instrument, using gene-specific sets of primers. The inflammatory gene expression levels obtained in an arbitrary unit (AU) were normalized to 18S as the housekeeping gene, and the relative data were calculated as the fold change.

### 2.15. Data Collection and Statistical Analyses

**Figure 1:**
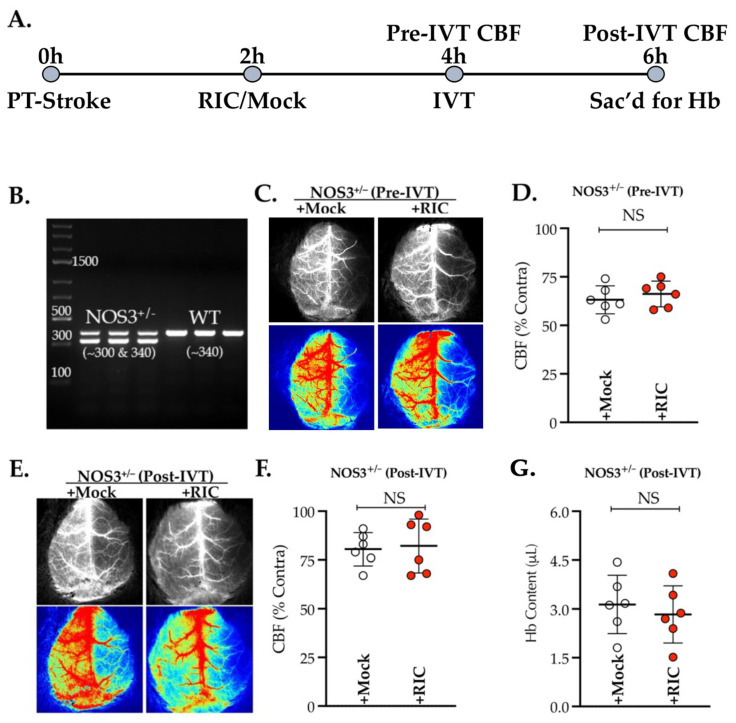
Genetic depletion of NOS3 abolishes the effect of RIC therapy. Male NOS3^+/^^−^ mice (10 ± 1 weeks old) were subjected to PT stroke, randomized, and treated with either RIC or RIC mock at 2 h post-stroke. Relative CBF was measured in all mice at 4 h post-stroke and also at 2 h after IVT therapy, i.e., 6 h post-stroke. (**A**) Schematic description of the experiment. (**B**) Representative gel image of PCR for genotype confirming the NOS3^+/^^−^ heterozygosity. (**C–F**) Representative LSCI maps (top greyscale) and pseudo-color images (bottom) analyzed for the relative quantitation of CBF, demonstrating no significant effect of RIC therapy. (**G**) Quantification of Hb at 6 h post-stroke also showed no significant effect of RIC in preventing the HT due to late IVT therapy. Data are presented as mean ± SD (*n* = 6 mice/group; *p* < 0.05; NS = not significant). No mortalities within this 6 h experiment occurred.

**Figure 2:**
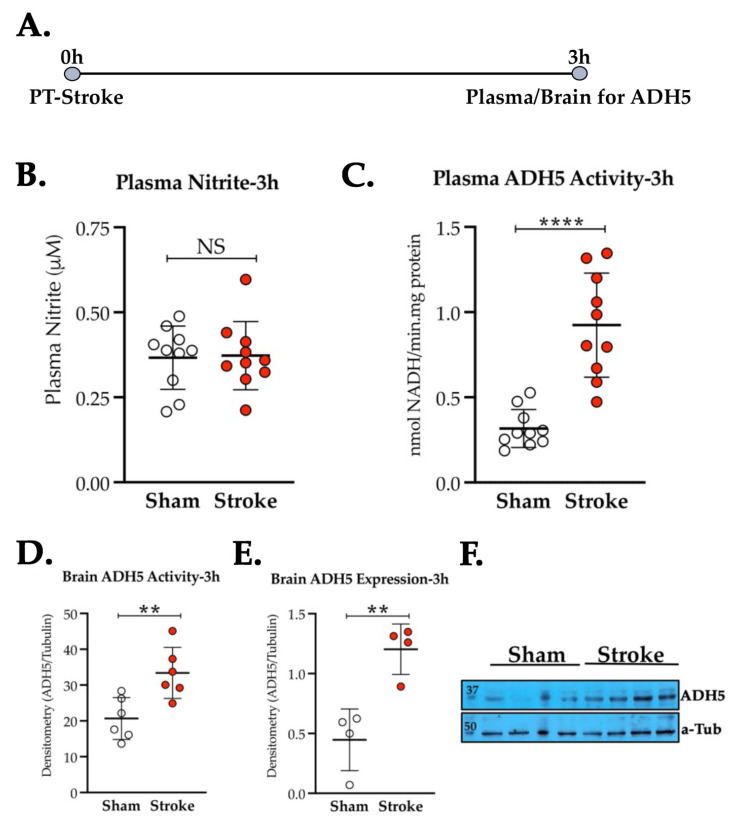
Effect of thrombotic stroke on the plasma NO_2_^−^ and ADH5/GSNOR in diabetic mice. Diabetic mice (14 ± 1 weeks old) were subjected to either PT stroke or sham-op. (**A**) Schematic description of the experiment. Blood for plasma and brains for BMECs were collected at 3 h post-stroke. (**B**) Plasma NO_2_^−^ level did not change significantly after stroke as compared to the sham-op diabetic group (*n* = 10 mice/group). (**C**,**D**) Thrombotic stroke significantly increased (**, *p* = 0.0001, ****, *p* = 0.0001) the activity of ADH5/GSNOR in plasma (*n* = 10 mice/group) and also in BMECs (*n* = 6 mice/group). (**E**,**F**) Stroke also increased the protein expression of ADH5/GSNOR in BMECs significantly (**, *p* = 0.0001) as compared to diabetic sham-op controls (*n* = 4 mice/group). Data are presented as mean ± SD (*p* < 0.05; NS = not significant). No mortalities occurred within 3 h of stroke.

**Figure 3:**
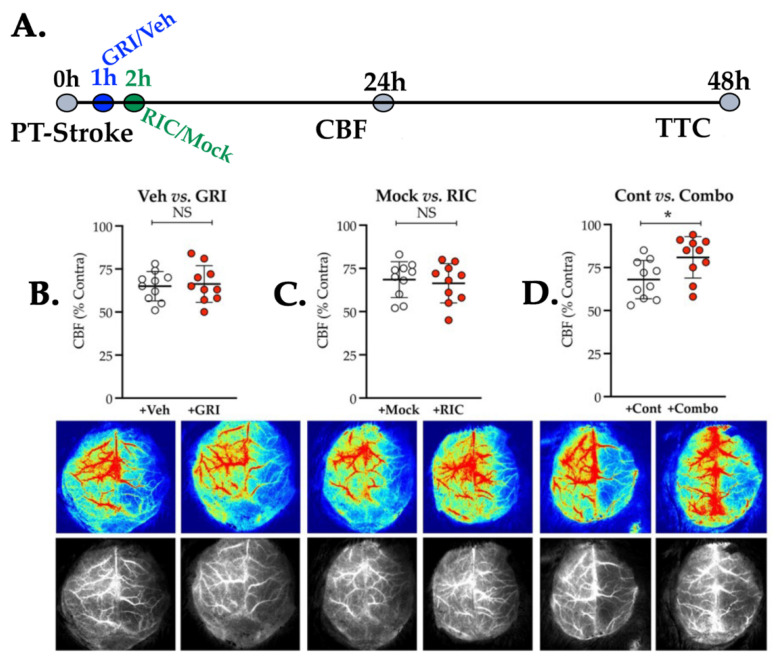
Effect of GRI, RIC, and their combination therapies on CBF in diabetic thrombotic stroke. (**A**) Schematic description of the experiment. Briefly, diabetic mice were subjected to PT stroke (*n* = 10 mice/group), followed by GRI therapy (+GRI) or vehicle infusion (+Veh) at 1 h post-stroke; RIC therapy (+RIC) or mock-op (+Mock) at 2 h post-stroke; and their combination (+Combo). (**B**,**C**) Relative CBF (top plots) and representative images (bottom) at 24 h post-stroke demonstrate that neither GRI nor RIC therapy alone significantly improved the CBF as compared to their diabetic stroke controls. (**D**) Combination therapy significantly improved (*, *p* = 0.011) the CBF as compared to the diabetic stroke controls, demonstrating that prior GRI treatment restores the efficacy of RIC to improve CBF in diabetic stroke. The data are presented as mean ± SD (*p* < 0.05; NS = not significant). No mortalities seen within 24 h of this mini-infarct stroke.

**Figure 4:**
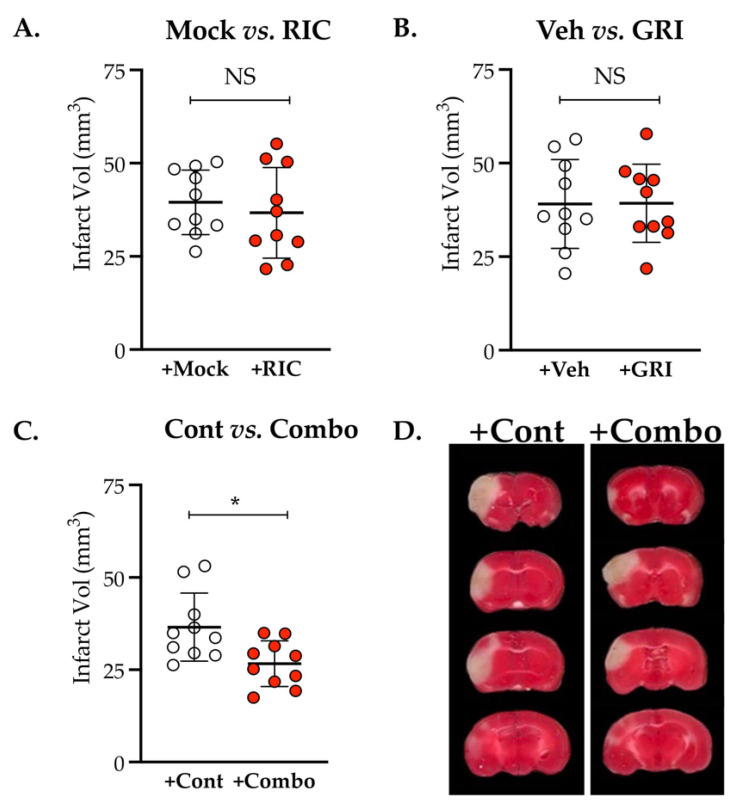
Effect of GRI therapy on restoring the neuroprotective efficacy of RIC therapy in diabetic thrombotic stroke. Diabetic mice, after in vivo CBF recording as described in Figure 3, were followed further for survival up to 48 h post-stroke. (**A**,**B**) Estimation and analysis of absolute infarct volume (in mm^3^) for both therapies given alone demonstrate that neither GRI nor RIC therapy alone significantly prevented the infarct progression following thrombotic stroke in diabetic mice as compared to their respective stroke controls. (**C**,**D**) A GRI+RIC combination therapy significantly attenuated the infarct progression as compared to the stroke controls (*, *p* = 0.011). Data are presented as mean ± SD (*p* < 0.05; NS = not significant). No mortalities occurred.

**Figure 5:**
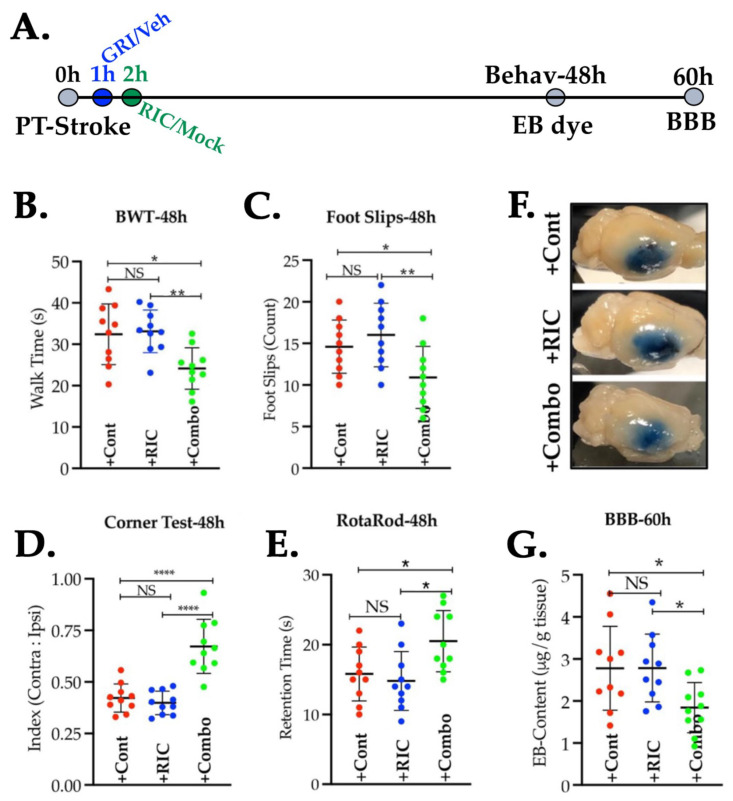
Prior GRI treatment following thrombotic stroke improves the neurobehavioral and neurovascular protection by RIC therapy in diabetic mice. (**A**) Schematic description of the experiment. Briefly, diabetic mice (*n* = 10 mice/group) pre-trained for behavioral tests were subjected to PT stroke, treatments, behavioral tests at 48 h, and thereafter BBB assessment at 60 h post-stroke. (**B**,**C**) BWT performances ((**B**) walk time: *, *p* = 0.012; ** *p* = 0.006; and (**C**) foot slips: *, *p* = 0.014; ** *p* = 0.001), (**D**) laterality index by corner test, and (**E**) motor coordination with rotarod test were not improved by the RIC therapy alone but were significantly by the GRI+RIC combination therapy as compared to the stroke controls and also as compared to the RIC alone group (Laterality Index: +Combo vs. +Cont ****, *p* = 0.0001, and +Combo vs. +Cont ****, *p* = 0.0001. Rotarod: +Combo vs. +Cont *, *p* = 0.045, and +Combo vs. +Cont, *, *p* = 0.013). (**F**,**G**) Representative images of perfused brains and the quantification of leaked EB dye in the brain after stroke, demonstrating that GRI+RIC combination therapy but not RIC alone significantly attenuated the BBB leakage as compared to the stroke control and RIC-alone groups (*, *p* = 0.041). Data are presented as mean ± SD (*p* < 0.05; NS = not significant).

**Figure 6:**
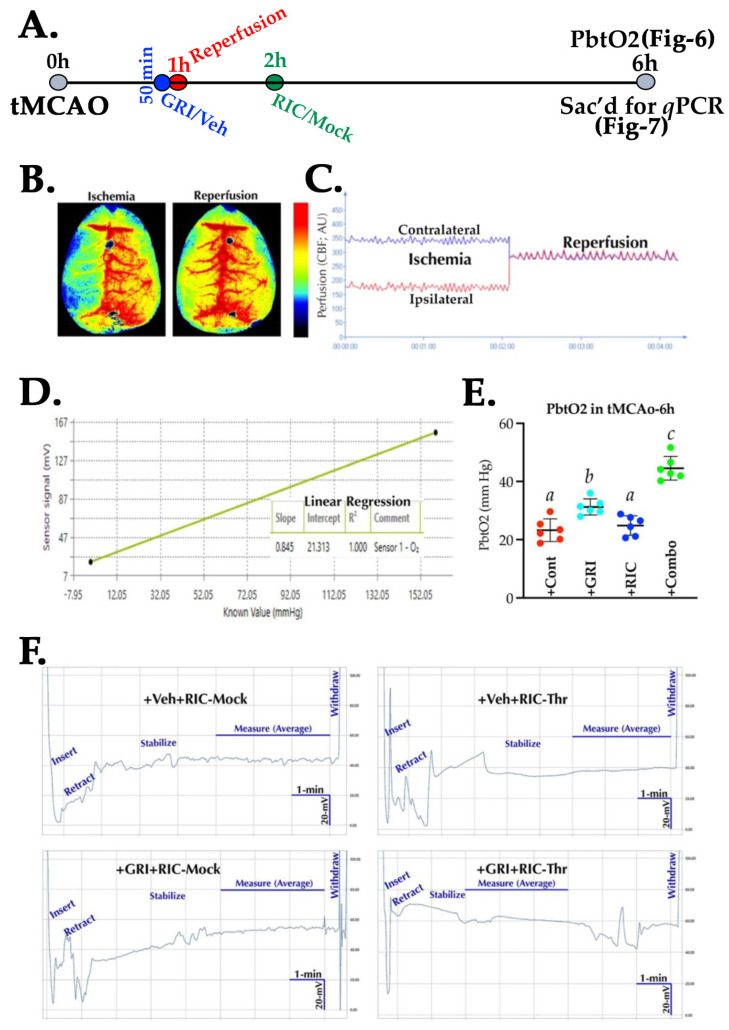
Effect of GRI and RIC therapy on brain tissue oxygenation in reperfused diabetic stroke. (**A**) Schematic description of the experiment. Briefly, diabetic mice (*n* = 6 mice/group) were subjected to tMCAO stroke followed by therapies as indicated. (**B**,**C**) A representative LSCI image and real-time perfusion curve showing successful occlusion–reperfusion. (**D**) A calibration curve and regression equation to calculate PbtO_2_. (**E**) Data plot demonstrating that GRI therapy alone improved PbtO_2_ significantly as compared to the stroke controls and RIC alone groups. RIC therapy alone did not improve PbtO_2_ despite reperfusion as compared to the stroke controls. Combination therapy augmented the benefits of reperfusion by increasing post-reperfusion PbtO_2_ as compared to all other 3 groups. (**F**) Representative real-time electrogram traces of each group, showing PbtO_2_ recording during a stabilized signal output period of 3 min. Data are expressed as mean ± SD. Pairs of means indicated with different alphabets are significantly different (*p* < 0.05).

**Figure 7:**
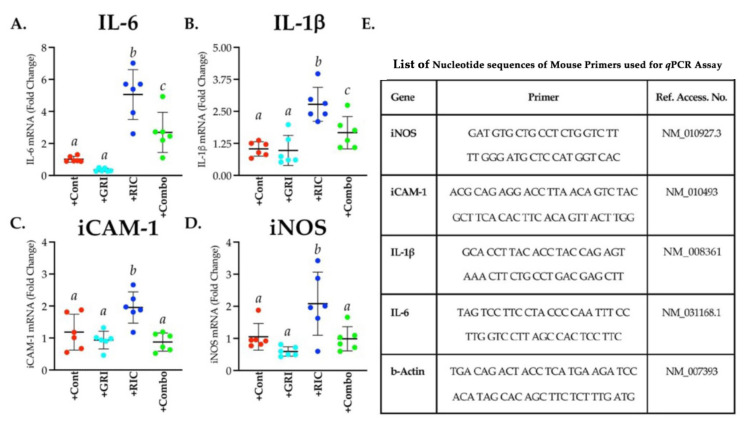
Effect of GRI, RIC, and their combination therapies on the early inflammatory gene expressions in diabetic mice subjected to tMCAO stroke. Brain samples from the mice used for the terminal PbtO_2_ measurement, as described in Figure 6 (*n* = 6 mice/group), were immediately harvested and analyzed for (**A**) IL-6, (**B**) IL-1β, (**C**) iCAM-1, and (**D**) iNOS. (**E**) List containing details of primer sets used for the qPCR assay. GRI therapy alone showed an insignificant trend only towards the downregulation of 3 out of 4 inflammatory genes. RIC therapy alone significantly enhanced the expressions compared to the other 3 groups. However, combination therapy significantly downregulated and prevented the RIC-induced early inflammatory responses as compared to the RIC therapy alone group. Data are expressed as mean ± SD, and the pairs of means indicated with different letters are significantly different (*p* < 0.05).

**Figure 8:**
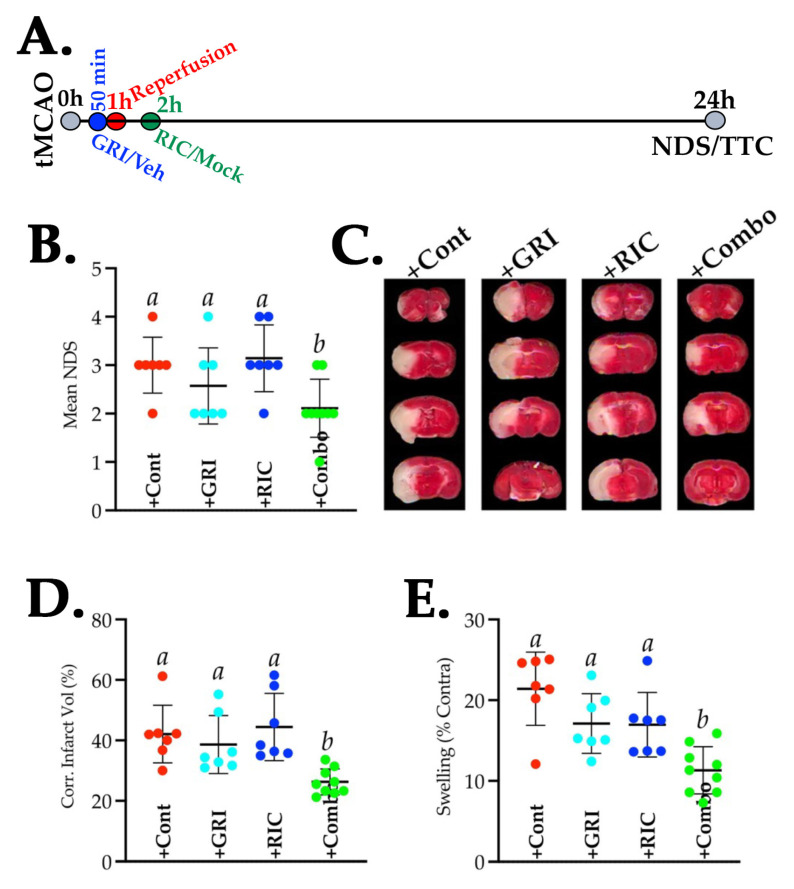
Effect of GRI, RIC, and their combination therapies on the neurofunctional outcomes following tMCAO stroke in diabetic mice. (**A**) Schematic description of the experiment. Briefly, diabetic mice (*n* = 10 mice/group) were subjected to the tMCAO model and were followed up to 24 h survival. Surviving mice (*n* = 7–9 mice/group) were assessed for the (**B**) neurological deficit score (NDS). (**C**–**E**) Representative TTC-stained images, infarct volume, and edema (% swelling) analyzed from images. GRI and RIC therapies alone did not attenuate neurological deficiency, stroke injury, and edema as compared to the stroke controls. All 3 outcome measures were significantly improved with the combination therapy as compared to the other 3 groups. Data are expressed as mean ± SD, and pairs of means indicated with different letters are significantly different (*p* < 0.05). Mortalities by 24 h were seen across all 4 groups in this tMCAO model of severe stroke injury (survival/mortality ratio: 7/3 (+Cont group); 7/3 (+GRI group); 7/3 (+RIC group); and 9/1 (+Combo group).

All the statistical analyses were performed using GraphPad Prism 9, GraphPad Software, San Diego, CA, USA). To estimate the sample size, a priori power analysis was performed based on our experiences and on historic data to achieve at least 80% power at 𝛼 = 0.05. The quantitative data are presented as mean ± SD, and statistical significance was determined at a *p*-value < 0.05. An unpaired t-test was performed to compare the two groups (Figure 1, Figure 2, Figure 3 and Figure 4). One-way analysis of variance (ANOVA) was used to compare the behavioral outcomes and the BBB permeability of the control group vs. the other 2 groups (Figure 5). In the experiments with a 2 × 2 factorial design (Figure 6, Figure 7 and Figure 8), a factorial ANOVA followed by post hoc comparisons was used to analyze the outcomes, as reported earlier [20]. No outlying data nor were any surviving animals excluded.

## 3. Results

### 3.1. NOS3 Depletion Abolishes the Efficacy of RIC Therapy in Thrombotic Stroke

We first tested the efficacy of RIC therapy given at 1 h post-thrombotic stroke in NOS3^+/−^ mice (Figure 1A), an animal model of preexisting NOS3 and endothelial dysfunctions. As anticipated, the partial depletion of NOS3 abolished the acute effect of the RIC therapy in enhancing CBF, as measured at 4 h post-stroke (*p* = 0.470; Figure 1B,C). Moreover, there was no significant effect of the RIC therapy later at 6 h post-stroke, i.e., 2 h after IVT therapy, in further enhancing the post-thrombolysis cerebral perfusion (*p* = 0.806; Figure 1D,E). The Hb content, a sign of the risk of hemorrhagic transformation (HT) due to late IVT therapy, also remained unchanged between the groups with or without RIC therapy at 6 h post-stroke (*p* = 0.565; Figure 1F). Thus, our data (Figure 1) demonstrate that NOS3/endothelial dysfunctions abolish the benefits of RIC therapy, at least in the early phase of thrombotic stroke, with or without IVT.

### 3.2. Thrombotic Stroke in Diabetic Mice Does Not Modulate the Plasma NO_2_^−^ Level but Increases the Activity and Expression of ADH5/GSNOR

Next, we determined the plasma NO_2_^−^ level as well as the enzymatic activity of ADH5/GSNOR, which enzymatically degrades and depletes the endogenous pool of SNO, another major component of bioactive NO [13,15]. As evident from Figure 2A, the plasma NO_2_^−^ level did not change significantly at 3 h post-thrombotic stroke in diabetic mice when compared to the sham-op diabetic group (*p* = 0.893). However, the activity of GSNOR was strongly upregulated at 3 h post-stroke in both the plasma samples (Stroke vs. Sham, *p* = 0.0001, Figure 2B) and in the BMECs (Stroke vs. Sham, *p* = 0.007, Figure 2C). Moreover, at the same time point, the GSNOR protein expression was also significantly increased in the BMECs of the stroked mice as compared to the sham-op group (Stroke vs. Sham, *p* = 0.004, Figure 2D,E).

### 3.3. Neither GRI nor RIC Therapy Alone but a Combination of Both Enhances CBF Following Thrombotic Stroke in Diabetic Mice

Next, we tested two endogenous NO-modulating therapies, GRI and RIC, in diabetic stroke (Figure 3). As anticipated, diabetes abolished the benefits of both the GRI and the RIC therapies in enhancing CBF, as measured at 24 h post-stroke, and thereby no significant vasculoprotective effects when given alone (+GRI vs. Veh, *p* = 0.767; and +RIC vs. +Mock, *p* = 0.671; Figure 3A,B). Interestingly, a combination of GRI and RIC therapies significantly enhanced the CBF (+Combo vs. +Cont, *p* = 0.023; Figure 3C).

### 3.4. Combination Therapy of GRI and RIC but Neither Therapy Alone in Diabetic Mice Is Neuroprotective against Thrombotic Stroke

We also assessed the neuroprotective (infarct reduction) effects of both the GRI and the RIC therapies individually and in combination at 48 h post-thrombotic stroke in the above-mentioned batch of diabetic mice. As is evident from our data, neither the GRI therapy (*p* = 0.971) nor the RIC therapy alone (*p* = 0.561) prevented the infarct size progression as compared to their respective controls (Figure 4A,B). However, the GRI–RIC combination therapy significantly attenuated the infarct progression as compared to their stroke controls (*p* = 0.011; Figure 4C), which agrees with the enhanced CBF effects of the combination therapy-treated group (Figure 3C).

### 3.5. Combination of GRI and RIC Therapies but Not RIC Alone in Thrombotic Diabetic Stroke Improves Behavioral Outcomes and Attenuates BBB Disruption

We next sought to know whether RIC alone or its combination with GRI improves behavioral outcomes after stroke via enhanced neurovascular protection (Figure 5A,B). RIC therapy alone, as compared to the stroke control group given the vehicle and the RIC mock, did not significantly improve the functional outcomes as tested with BWT in the context of walk time (*p* = 0.959) and also in the context of the number of foot slips (*p* = 0.614). On the other hand, the combination therapy group showed a significantly improved post-stroke walk time (*p* = 0.012) and also a reduced number of foot slips (*p* = 0.014) as compared to the stroke controls and also as compared to the group treated with RIC alone (walk time, *p* = 0.006; and foot slips, *p* = 0.001).

Moreover, the laterality index, as examined with the corner test, was improved significantly in the combination therapy group as compared to the stroke controls, as well as to the groups treated with RIC alone (*p* = 0.0001; Figure 5C), but not when compared between the +RIC alone vs. +Cont groups (*p* = 0.836). Similarly, the motor coordination outcome measure with the rotarod test was also significantly improved in the combination therapy group as compared to the stroke control group (*p* = 0.045) and also as compared to RIC therapy alone (*p* = 0.013). However, RIC treatment alone remained ineffective when compared to the stroke control group (*p* = 0.853; Figure 5D). These mice, when tested for the degree of BBB leakage, also showed similar outcomes (Figure 5E,F). A combination of the GRI and RIC therapies in diabetic thrombotic stroke significantly attenuated the disruption of BBB at 60 h post-stroke as compared to both the stroke control group and the groups treated with RIC alone (*p* = 0.041), while the RIC therapy alone was not effective in reducing the BBB leakage in diabetic stroke as compared to the control group (*p* = 0.994). 

### 3.6. GRI and RIC Therapies Show Differential Effects on PbtO_2_ in Reperfused Diabetic Stroke

We next tested the GRI alone, the RIC alone, and their combination therapy in the reperfused tMCAO model (Figure 6A,B) to assess the benefits of these treatments on brain tissue reoxygenation. Unlike the thrombotic stroke, GRI therapy alone in reperfused diabetic stroke significantly improved the PbtO_2_ level as compared to the stroke control group (*p* = 0.010) and also as compared to the RIC alone group (*p* = 0.028), while RIC therapy alone did not show any significant effects as compared to the stroke controls (*p* = 0.853; Figure 6D,E). However, RIC therapy in combination with GRI treatment in diabetic stroke further enhanced the PbtO_2_ significantly as compared to all the other three groups, GRI, RIC, and stroke controls (*p* = 0.0001), demonstrating that prior GRI treatment improves the benefits of RIC therapy in reperfusion.

### 3.7. RIC Therapy Triggers Inflammatory Gene Expressions in Reperfused Diabetic Stroke which Is Attenuated in Combination with GRI Treatment

As evident from our data, RIC therapy alone in diabetic stroke unexpectedly augmented the inflammatory gene expressions significantly as compared to the stroke control group when analyzed for IL-6 (*p* = 0.0001), IL-1β (*p* = 0.0002), iCAM-1 (*p* = 0.030), and iNOS (*p* = 0.021) at 6 h post-stroke (Figure 7). Moreover, GRI treatment alone did not show any significant effects on the above four inflammatory gene expressions as compared to the stroke control group. A combination of GRI treatment with RIC therapy significant suppressed these inflammatory responses as compared to the RIC alone group (IL-6, *p* = 0.005; IL-1β, *p* = 0.011; iCAM-1, *p* = 0.003; and iNOS, *p* = 0.014). However, these inflammatory responses did not change and reduce to the basal level despite the combination therapy.

### 3.8. GRI Treatment in Combination with RIC Therapy Attenuates Reperfusion Injury and Enhances the Neuroprotection in Diabetic Stroke

Lastly, a set of diabetic stroke mice with or without GRI and RIC therapies were followed for 24 h post-tMCAO to assess the benefits of these therapies in attenuating reperfusion injury and outcomes (Figure 8). As is evident from our data, neither a single dose of GRI (*p* = 0.595) nor the one-time RIC (*p* = 0.974) therapies in diabetic mice attenuated the neurologic deficit when compared to the stroke control group at 24 h post-stroke (Figure 8A). Instead, a combination of both therapies in diabetic stroke significantly attenuated the neurologic deficit as compared to the stroke controls (*p* = 0.039) and RIC alone as well (*p* = 0.016). Moreover, combination therapy also reduced the infarct volume significantly (Figure 8B,C) as compared to stroke control (*p* = 0.007), GRI treatment alone (*p* = 0.036), and RIC therapy alone (*p* = 0.002), which also translated into significantly reduced edema (Combo vs. Cont, *p* = 0.0003; vs. GRI treatment alone, *p* = 0.024; and vs. RIC therapy alone, *p* = 0.0281; Figure 8D). On the other hand, the effects of individual therapies remained statistically not significant in modulating the infarct volume and edema as compared to both the stroke controls and the combination therapy groups.

## 4. Discussion

RIC therapy is an emerging intervention for NOS3 activation in IR injuries [16,18,22,45,46]. In the present work, we tested RIC therapy in well-accepted preclinical settings of decreased NOS3 and comorbidity, reporting a translationally critical finding in stroke models that endothelial dysfunction and comorbidity abolish the benefits of RIC therapy. We also report that pharmacological inhibition of ADH5/GSNOR, as an adjunct treatment to RIC therapy, turns RIC protective in diabetic stroke. Our report carries invaluable translational information on comorbid stroke to improve the efficacy of RIC, a promising therapy with neutral effects so far in stroke clinical trials [47,48].

The molecular mechanism of RIC-mediated neurovascular protection could be multifactorial, including increased NO to improve tissue perfusion [16,49,50]; yet, RIC therapy remains understudied in the animal models of comorbid stroke with impaired NOS3 activity, although the loss of NOS3 abolished the neuroprotective benefits of IC in mechanical IR models [18,46]; whether this loss of benefits was due to no effects on improving CBF remains unknown in thromboembolic stroke models, the most common type of strokes in humans [51]. Herein, instead of utilizing a genetic model of complete NOS3 deletion, we first tested RIC therapy in the partially depleted NOS3 mutant (NOS3^+/−^; Figure 1A) mice, which more appropriately modeled a scenario of NOS3-associated vascular dysfunction. As evident from our data (Figure 1B,C), NOS3 depletion abolished the early benefits of RIC in enhancing CBF. Moreover, unlike our prior reports in WT mice without comorbidity [20,21], this loss of effect of RIC therapy on CBF and the prevention of HT was also not evident despite reperfusion with IVT (Figure 1D,E). Thus, we demonstrated that NOS3 activation by RIC during stroke is an essentially required to enhance the CBF.

Developing countries such as the Kingdom of Saudi Arabia (KSA) and also the developed countries are now witnessing an escalation in the rate of metabolic disorders [51,52,53,54]. Diabetes, often accompanied by endothelial dysfunctions and a poor level of bioactive NO, is one such disorder that increases the risk of stroke by at least twofold [52,53] and is known to increase the risk and severity of stroke [10,55]. The low level of NO_2_^−^, the most common form of endogenous bioactive NO, correlated with the increased severity, mortality, and poor outcomes after stroke [56]. Unexpectedly, we did not find any significant decrease in the plasma NO_2_^−^ level in diabetic stroke mice (Figure 2A), a well-accepted animal model for studying comorbid stroke [57]. Thus, our finding indicates that plasma NO_2_^−^ may not be the primary NO modulator in mediating the early phase neurovascular protection by endogenous NO in diabetic stroke [2,12]. Notably, we reported in diabetic thrombotic stroke that supplementation of exogenous GSNO, the major alternate endogenous NO reservoir and the prime catabolic target of GSNOR, enhances CBF and improves brain tissue reoxygenation, a true translatory signature of increased microvascular reperfusion in deep brain tissues [2,12,57]. Others have also reported that GSNO in rodents and humans attenuates IR injury, embolization, and secondary ischemia [14,58,59,60] and induces hypoxic vasodilation to enhance CBF [4,12]. Interestingly, the activity of GSNOR significantly increased in the early hours of acute stroke (Figure 2B–E), indicating a possible role of GSNOR in post-stroke impaired microcirculatory reflow via degradation of SNO [15]. Therefore, it drew our collective attention to inhibit GSNOR prior to RIC to preserve the RIC-mediated bioactive SNOs to improve CBF and PbtO_2_.

Prior studies reported that both GRI and RIC therapies alone are protective in mechanical IR models of stroke in mice without comorbidities [14,15,18,22,46]. While the genetic deletion or pharmacological inhibition of GSNOR elevated the endogenous level of SNO/GSNO to protect against IR injury [13], RIC therapy also conferred protection via enhanced *s*-nitrosylation [23]. Moreover, both the genetic deletion of NOS3 and hyperglycemia (a state of impaired NOS3) abolished the benefits of IC [18,24,25,26,46,61]. As such, an elevated level of glycosylated Hb in diabetes scavenges and tightly bounds with NO to attenuate its bioactivity [30], which likely may result in augmented endothelial dysfunctions. As a large number of stroke patients are presented with comorbidities accompanied by endothelial dysfunctions, the net effect of RIC may be neutralized in the patient cohorts of the clinical trial carrying a mixed population with or without comorbidities. Hence, the recent clinical trials in stroke found no benefits but instead neutral effects of RIC therapy [47,48]. Therefore, it is essential to test and validate RIC therapy in comorbid strokes and develop strategies to turn it protective via the preservation and bioavailability of SNO reservoirs. When we tested GRI and RIC therapy in diabetic mice following thrombotic stroke, both remained ineffective when alone to improve the CBF and to reduce the stroke injury. However, a combination of both showed significant protection in enhancing the CBF and also in decreasing the stroke injury (Figure 3 and Figure 4). Thus, we envision that RIC therapy in diabetic mice might be effective in generating the bioactive NO in the form of SNO reservoirs. However, increased GSNOR activity could be effective in catabolizing SNOs to neutralize the therapeutic benefits of RIC. We further confirmed it by testing the RIC with or without GRI where the combination therapy again promoted neurobehavioral recovery and conferred neurovascular protection by preserving the BBB integrity (Figure 5), but the RIC alone remained ineffective. Thus, we demonstrate that stroke-associated comorbidity accompanying NOS3 dysfunctions may depreciate the early phase benefits of RIC in thrombotic stroke, which needs to be carefully investigated in clinical trials.

Animal models of stroke often differ by the virtue of CBF restoration, and these dynamic changes in CBF may affect the efficacy of therapies [62]. A large number of stroke patients are now being treated with endovascular thrombectomy (EVT), resulting in a sudden large vessel reperfusion which may also cause reperfusion-associated injury [63], a fashion of CBF restoration very different to the spontaneous or IVT-mediated slower reperfusion [62]. Therefore, we also tested GRI treatment in combination with or without RIC therapy in reperfused diabetic stroke. As is evident from our data, again the RIC alone in diabetic stroke was not effective in enhancing brain tissue reoxygenation and failed to reduce the infarct volume size despite reperfusion (Figure 6 and Figure 8). Surprisingly, in this reperfused model, GRI therapy alone enhanced the early phase PbtO_2_ at 3 h post-stroke. However, this single dose of GRI alone did not translate into reduced neurofunctional outcomes later at 24 h post-stroke. This discrepancy in not reducing the infarct size by GRI in our finding and the prior report could be attributed to the differences in the dose of GRI used [14]. While we used only 2.5 mg/kgbwt of GRI in this tMCAO study based on our dose-standardization in the thrombotic stroke model (data not shown here), others used 5 mg/kgbwt, likely giving a longer window of protection by GRI [14]. The use of a higher dose or the repeated lower dose of GRI in future studies may resolve this discrepancy. Moreover, RIC alone unexpectedly augmented the expression of various early phase inflammatory genes in diabetic mice that were again downregulated in combination with GRI therapy but not by the GRI treatment alone (Figure 7). However, this effect of RIC in enhancing the acute inflammatory genes at 6 h post-stroke may be transient but not detrimental as we have not seen any unwanted effects of RIC despite the comorbidity.

## 5. Conclusions

In conclusion, we demonstrate that preexisting endothelial dysfunctions and comorbidity abolish the benefits of RIC therapy, and therefore, in future clinical trials, different regimens of RIC should be tested and manipulated for enhanced efficacy in comorbid stroke. Furthermore, GRI therapy alone may also have neutral effects in comorbid strokes, which needs to be further investigated. Owing to the NO metabolome modulating effects of the GRI and RIC therapies [13,15], a combination treatment could be beneficial in comorbid and, in particular, diabetic stroke. Notably, we also acknowledge certain caveats in our present study. First, we have not been able to directly measure the effect of RIC and GRI on SNO/GSNO content as it is extremely tricky and requires state-of-the-art technique which we currently lack in our laboratory. However, both are well known to act via preservation and enhancement of the SNO/GSNO pool [13,15]. Second, as stroke is prevalent in aged individuals and is often accompanied by metabo-physiological disorders, this GRI–RIC combination therapy needs to be tested in aged diabetic and hypertensive animals for both short- and long-term outcomes. Third, as GSNOR may have a gender-dependent activity, this combination therapy also should be tested following comorbid strokes in both sexes. We anticipate that our ongoing work to develop an affordable quick method of assay for SNO/GSNO will possibly help to translate a reliable blood-associated biomarker to monitor the response and efficacy of RIC therapy in ischemic disorders.

## 6. Patents

SKZ holds a patent for the use of GRI in combination with RIC therapy in comorbid stroke (2021 US Patent 11071733).

## Figures and Tables

**Figure A1:**
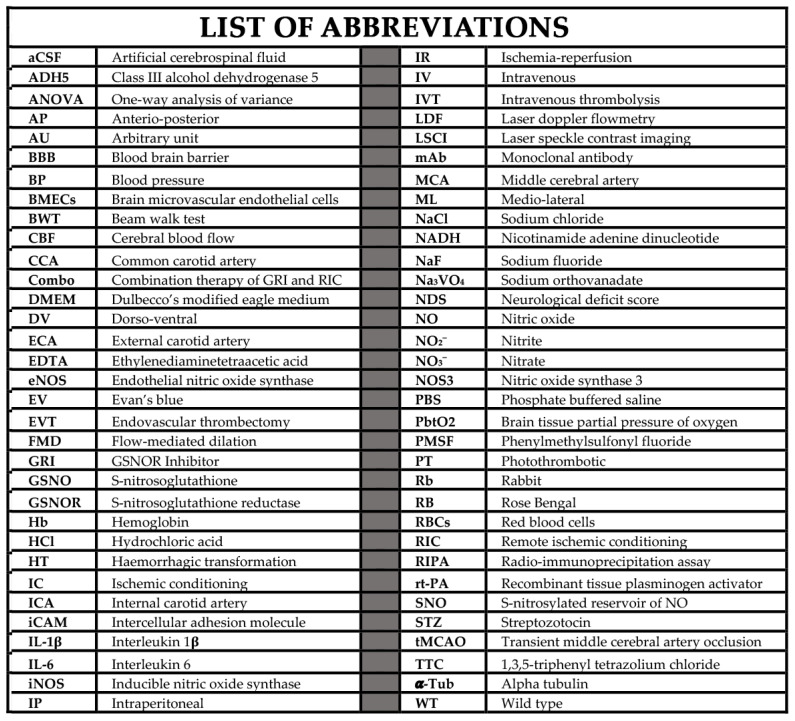
List of Abbreviations.

## Data Availability

The data presented in this study are available with the corresponding author, and not publicly available immediately due to ongoing intellectual property interest.
